# Supplementary material for: A Likelihood Approach for Real-Time Calibration of Stochastic Compartmental Epidemic Models
Source: PLoS Comput Biol. 2017 Jan 17;13(1):e1005257. doi: 10.1371/journal.pcbi.1005257 (PMC5240920; doi:10.1371/journal.pcbi.1005257)
Supplement: S1 File — (TAR.GZ) [file pcbi.1005257.s014.tar.gz › HSPH_Online-SI-Revision/EnKF07/n10-severe/n10-severe_EnKF2_table-4.pdf]

1	newly 3 weeks spec	606.	206.79	{0., 136.}	108.761	2.13681	newly 3 weeks cumul	1634.	1428.7	{53., 6833.}	126.682	0.927472	newly 5 weeks spec	589.	78.36	{0., 73.}	94.0102	2.31352	newly 5 weeks cumul	2883.	1636.33	{66., 6858.}	108.332	1.05351	newly 55 weeks cumul	5530.	1772.07	{72., 6858.}	92.181	1.21007
2	newly 3 weeks spec	693.	72.15	{0., 84.}	93.5945	2.04923	newly 3 weeks cumul	1675.	1235.47	{91., 1995.}	116.206	0.855451	newly 5 weeks spec	711.	25.55	{0., 47.}	97.4754	2.79637	newly 5 weeks cumul	3118.	1295.44	{91., 2070.}	99.0648	1.02645	newly 57 weeks cumul	5958.	1413.31	{91., 2070.}	90.6145	1.19721
3	newly 3 weeks spec	672.	259.65	{0., 767.}	91.7842	1.51575	newly 3 weeks cumul	1714.	1652.71	{23., 7749.}	131.368	0.933249	newly 5 weeks spec	640.	60.01	{0., 41.}	94.9266	2.43139	newly 5 weeks cumul	3081.	1832.02	{27., 7927.}	103.607	1.06271	newly 59 weeks cumul	4699.	1952.76	{30., 7938.}	89.6327	1.15601
4	newly 3 weeks spec	729.	151.1	{34., 217.}	88.7984	0.94736	newly 3 weeks cumul	1838.	587.38	{135., 781.}	81.5713	0.717295	newly 5 weeks spec	670.	80.63	{17., 126.}	90.1925	1.20841	newly 5 weeks cumul	3254.	779.85	{183., 1092.}	85.3396	0.843523	newly 54 weeks cumul	5574.	1043.73	{241., 1752.}	85.9851	0.955115
5	newly 3 weeks spec	577.	149.37	{42., 173.}	87.3293	0.841294	newly 3 weeks cumul	1549.	570.28	{156., 694.}	78.4248	0.624461	newly 5 weeks spec	572.	82.97	{17., 140.}	90.1031	1.16194	newly 5 weeks cumul	2731.	767.01	{207., 955.}	83.4782	0.755297	newly 54 weeks cumul	5177.	1049.94	{325., 1768.}	85.407	0.914096
6	newly 3 weeks spec	667.	155.45	{0., 151.}	102.043	2.39957	newly 3 weeks cumul	1499.	1201.21	{26., 3231.}	107.738	0.844783	newly 5 weeks spec	615.	76.82	{0., 57.}	96.5008	2.52334	newly 5 weeks cumul	2818.	1388.82	{32., 4656.}	97.4074	1.01163	newly 55 weeks cumul	4913.	1544.15	{41., 4883.}	89.8817	1.14961
7	newly 3 weeks spec	404.	148.97	{52., 240.}	79.9678	0.660992	newly 3 weeks cumul	1214.	554.24	{248., 967.}	69.4399	0.502002	newly 5 weeks spec	360.	90.68	{12., 127.}	87.7056	0.940703	newly 5 weeks cumul	2017.	763.21	{322., 1291.}	75.5325	0.602215	newly 54 weeks cumul	3462.	1119.54	{403., 1647.}	82.186	0.715811
8	newly 3 weeks spec	687.	101.1	{7., 327.}	86.5386	1.20816	newly 3 weeks cumul	1740.	552.04	{41., 841.}	81.1425	0.866596	newly 5 weeks spec	686.	42.47	{1., 90.}	93.809	1.74871	newly 5 weeks cumul	3148.	657.35	{58., 1539.}	82.8409	1.01693	newly 58 weeks cumul	4943.	787.5	{58., 2438.}	84.4127	1.13072
9	newly 3 weeks spec	655.	183.09	{13., 705.}	80.7496	0.974761	newly 3 weeks cumul	1488.	2352.97	{919., 6011.}	91.4671	0.25637	newly 5 weeks spec	787.	39.95	{0., 80.}	94.9238	2.45799	newly 5 weeks cumul	3066.	2476.66	{931., 6020.}	63.8213	0.360072	newly 62 weeks cumul	5852.	2512.27	{933., 6021.}	62.8587	0.52547
10	newly 3 weeks spec	791.	182.39	{9., 369.}	84.3047	1.03591	newly 3 weeks cumul	2024.	2334.79	{893., 5705.}	79.3958	0.30513	newly 5 weeks spec	740.	44.37	{0., 64.}	95.9797	2.26526	newly 5 weeks cumul	3581.	2460.61	{934., 6047.}	69.5373	0.424387	newly 62 weeks cumul	5507.	2527.82	{937., 6150.}	69.2083	0.544417
11	newly 3 weeks spec	764.	56.44	{0., 66.}	96.0052	2.43465	newly 3 weeks cumul	1667.	979.85	{159., 1716.}	76.2669	0.565613	newly 5 weeks spec	893.	17.61	{0., 13.}	98.028	3.12519	newly 5 weeks cumul	3478.	1028.79	{180., 1716.}	79.6317	0.813439	newly 61 weeks cumul	4839.	1050.56	{181., 1820.}	82.8911	0.939948
12	newly 3 weeks spec	676.	50.91	{0., 78.}	96.2115	2.38897	newly 3 weeks cumul	1411.	751.56	{160., 1375.}	67.6499	0.510019	newly 5 weeks spec	750.	15.54	{0., 15.}	97.928	3.06838	newly 5 weeks cumul	2924.	794.09	{177., 1375.}	78.6508	0.774522	newly 61 weeks cumul	5377.	816.43	{180., 1384.}	85.7406	1.01809
13	newly 3 weeks spec	785.	108.53	{5., 347.}	87.5605	1.32878	newly 3 weeks cumul	1843.	614.42	{30., 1096.}	84.3407	0.938849	newly 5 weeks spec	871.	40.9	{1., 86.}	95.3042	1.92316	newly 5 weeks cumul	3619.	722.6	{36., 1640.}	83.4579	1.12459	newly 58 weeks cumul	6232.	798.62	{36., 2682.}	87.1852	1.29682
14	newly 3 weeks spec	790.	271.89	{5., 657.}	100.966	1.4871	newly 3 weeks cumul	2006.	858.78	{119., 2238.}	92.012	0.944072	newly 5 weeks spec	648.	113.74	{0., 627.}	89.1821	2.23917	newly 5 weeks cumul	3522.	1176.61	{124., 3565.}	91.098	1.11074	newly 60 weeks cumul	4964.	1344.7	{127., 6038.}	87.7051	1.21406
15	newly 3 weeks spec	973.	669.35	{89., 1973.}	65.2631	0.500855	newly 3 weeks cumul	2131.	4591.49	{1142., 10 879.}	144.95	0.379485	newly 5 weeks spec	967.	190.13	{5., 389.}	82.636	1.18211	newly 5 weeks cumul	4148.	5124.6	{1200., 11 469.}	76.8023	0.343869	newly 64 weeks cumul	6236.	5423.25	{1209., 11 656.}	58.9604	0.363679
16	newly 3 weeks spec	860.	331.04	{3., 1088.}	102.121	1.52088	newly 3 weeks cumul	2325.	871.05	{43., 2739.}	85.8766	0.990266	newly 5 weeks spec	767.	138.37	{0., 748.}	86.2516	2.25488	newly 5 weeks cumul	3984.	1249.75	{52., 4541.}	86.8722	1.14592	newly 60 weeks cumul	5671.	1472.71	{56., 6911.}	83.7269	1.24602
17	newly 3 weeks spec	584.	207.05	{3., 273.}	107.238	1.53846	newly 3 weeks cumul	1339.	556.96	{25., 1445.}	92.0822	0.911771	newly 5 weeks spec	581.	80.79	{0., 202.}	93.4578	2.46204	newly 5 weeks cumul	2523.	791.44	{27., 2178.}	93.0868	1.1146	newly 60 weeks cumul	5901.	879.6	{27., 2320.}	89.8346	1.42105
18	newly 3 weeks spec	574.	108.02	{5., 306.}	85.7561	1.22138	newly 3 weeks cumul	1527.	592.95	{31., 1058.}	84.8913	0.910787	newly 5 weeks spec	639.	42.93	{1., 84.}	93.3005	1.76193	newly 5 weeks cumul	2806.	702.11	{35., 1605.}	83.567	1.06894	newly 58 weeks cumul	5958.	790.98	{39., 2798.}	86.7489	1.31035
19	newly 3 weeks spec	718.	168.75	{50., 205.}	89.7618	0.914915	newly 3 weeks cumul	1880.	625.34	{246., 695.}	83.5436	0.694807	newly 5 weeks spec	617.	99.63	{15., 216.}	89.1945	1.16106	newly 5 weeks cumul	3238.	855.47	{317., 1113.}	86.2412	0.809837	newly 54 weeks cumul	5565.	1249.	{395., 2341.}	84.8579	0.918476
20	newly 3 weeks spec	409.	141.71	{20., 196.}	88.9071	0.851023	newly 3 weeks cumul	1135.	547.28	{91., 690.}	77.5648	0.62386	newly 5 weeks spec	432.	81.73	{8., 163.}	90.5208	1.2065	newly 5 weeks cumul	2027.	736.07	{118., 1000.}	82.8411	0.75755	newly 54 weeks cumul	5250.	1052.62	{151., 1804.}	87.0133	1.05369
21	newly 3 weeks spec	797.	186.86	{11., 407.}	83.8269	1.0433	newly 3 weeks cumul	1935.	2065.7	{442., 4528.}	76.8579	0.328783	newly 5 weeks spec	789.	45.5	{0., 69.}	95.9645	2.28876	newly 5 weeks cumul	3576.	2201.78	{519., 4765.}	69.0906	0.467897	newly 62 weeks cumul	5739.	2288.4	{538., 5019.}	71.3595	0.611048
22	newly 3 weeks spec	515.	195.2	{3., 277.}	108.322	1.54124	newly 3 weeks cumul	1274.	685.83	{21., 1336.}	101.867	0.947551	newly 5 weeks spec	507.	75.56	{0., 193.}	93.1874	2.36754	newly 5 weeks cumul	2335.	897.59	{24., 2103.}	96.8475	1.13041	newly 60 weeks cumul	4471.	1010.52	{24., 2193.}	91.5706	1.35805
23	newly 3 weeks spec	509.	158.59	{1., 640.}	93.5147	1.50586	newly 3 weeks cumul	1486.	1262.08	{28., 5464.}	127.168	0.925022	newly 5 weeks spec	479.	34.51	{0., 38.}	96.0772	2.44385	newly 5 weeks cumul	2515.	1362.9	{33., 5622.}	106.383	1.05837	newly 59 weeks cumul	4951.	1458.48	{33., 6527.}	89.8243	1.25274
24	newly 3 weeks spec	864.	245.58	{2., 380.}	101.352	1.64885	newly 3 weeks cumul	1807.	720.75	{18., 2083.}	94.2407	1.05956	newly 5 weeks spec	1016.	91.13	{0., 272.}	91.0305	2.43228	newly 5 weeks cumul	3920.	977.97	{20., 2217.}	91.6635	1.29901	newly 60 weeks cumul	6534.	1095.12	{20., 2677.}	88.4423	1.4629
25	newly 3 weeks spec	883.	307.31	{0., 768.}	77.3828	1.41066	newly 3 weeks cumul	1516.	4026.18	{744., 10 154.}	187.15	0.409463	newly 5 weeks spec	1165.	89.08	{0., 275.}	92.3536	2.33917	newly 5 weeks cumul	3941.	4270.33	{776., 10 779.}	69.3187	0.333914	newly 63 weeks cumul	5681.	4451.92	{778., 11 771.}	62.54	0.397119
26	newly 3 weeks spec	596.	128.17	{13., 170.}	87.1896	0.9989	newly 3 weeks cumul	1544.	2011.68	{544., 5727.}	80.0855	0.272856	newly 5 weeks spec	608.	30.61	{0., 35.}	97.8635	2.37862	newly 5 weeks cumul	2767.	2099.75	{548., 5729.}	69.6722	0.400013	newly 62 weeks cumul	4667.	2148.55	{548., 5729.}	69.7958	0.550494
27	newly 3 weeks spec	646.	273.61	{0., 726.}	75.0759	1.43911	newly 3 weeks cumul	1397.	3400.91	{730., 7426.}	163.228	0.377513	newly 5 weeks spec	776.	92.88	{0., 295.}	89.183	2.27875	newly 5 weeks cumul	2980.	3643.44	{743., 7426.}	64.4054	0.267026	newly 63 weeks cumul	6059.	3829.75	{744., 7426.}	55.0607	0.390112
28	newly 3 weeks spec	775.	135.88	{13., 159.}	89.2594	1.16779	newly 3 weeks cumul	1597.	1983.49	{467., 5537.}	77.3093	0.292966	newly 5 weeks spec	804.	37.18	{0., 28.}	97.1045	2.55552	newly 5 weeks cumul	3259.	2089.36	{471., 5543.}	65.9761	0.446235	newly 62 weeks cumul	5190.	2147.48	{471., 5543.}	68.1911	0.585242
29	newly 3 weeks spec	901.	591.57	{72., 1497.}	64.434	0.49551	newly 3 weeks cumul	1967.	4110.73	{718., 9349.}	141.202	0.388477	newly 5 weeks spec	1020.	172.81	{8., 384.}	83.6559	1.18323	newly 5 weeks cumul	4131.	4586.07	{925., 10 738.}	70.7139	0.3568	newly 64 weeks cumul	5712.	4859.22	{928., 11 393.}	60.0868	0.381552
30	newly 3 weeks spec	883.	544.72	{100., 1029.}	57.8913	0.437416	newly 3 weeks cumul	1653.	4001.21	{1182., 9247.}	158.678	0.36945	newly 5 weeks spec	1189.	158.	{7., 370.}	86.9218	1.2377	newly 5 weeks cumul	4088.	4437.2	{1276., 10 419.}	59.4721	0.283161	newly 64 weeks cumul	6316.	4701.77	{1285., 10 643.}	52.3602	0.337688
31	newly 3 weeks spec	575.	70.38	{0., 101.}	94.7757	2.31845	newly 3 weeks cumul	1530.	870.22	{173., 1374.}	74.8641	0.540478	newly 5 weeks spec	506.	24.01	{0., 44.}	97.0652	2.76229	newly 5 weeks cumul	2641.	936.56	{180., 1586.}	79.4199	0.726166	newly 61 weeks cumul	4464.	968.41	{186., 1717.}	83.5894	0.925044
32	newly 3 weeks spec	390.	166.67	{34., 227.}	86.4692	0.693068	newly 3 weeks cumul	1183.	626.48	{236., 855.}	79.1209	0.536879	newly 5 weeks spec	341.	96.64	{11., 157.}	87.2669	0.930384	newly 5 weeks cumul	1958.	849.78	{306., 1163.}	82.285	0.630845	newly 54 weeks cumul	3806.	1177.36	{387., 1841.}	84.33	0.779501
33	newly 3 weeks spec	499.	234.58	{52., 408.}	80.7816	0.67093	newly 3 weeks cumul	1415.	1148.68	{225., 1616.}	84.6558	0.479182	newly 5 weeks spec	521.	121.26	{8., 191.}	87.7735	1.09637	newly 5 weeks cumul	2457.	1435.34	{289., 2049.}	81.5547	0.584078	newly 54 weeks cumul	5031.	1781.06	{386., 4692.}	79.9833	0.757865
34	newly 3 weeks spec	526.	214.57	{47., 303.}	87.7205	0.732679	newly 3 weeks cumul	1521.	872.81	{202., 1358.}	83.426	0.565473	newly 5 weeks spec	469.	109.11	{14., 154.}	89.8571	1.02937	newly 5 weeks cumul	2539.	1136.57	{269., 1747.}	85.1158	0.666428	newly 54 weeks cumul	4810.	1429.43	{393., 2162.}	85.4019	0.806682
35	newly 3 weeks spec	688.	59.09	{0., 66.}	95.9724	2.2957	newly 3 weeks cumul	1586.	927.04	{160., 1872.}	71.5095	0.537005	newly 5 weeks spec	769.	18.54	{0., 15.}	97.5891	3.05822	newly 5 weeks cumul	3166.	979.17	{178., 1872.}	76.7047	0.765879	newly 61 weeks cumul	5296.	1002.07	{178., 2019.}	82.351	0.963413
36	newly 3 weeks spec	502.	74.1	{0., 93.}	93.1594	1.86976	newly 3 weeks cumul	1350.	1133.11	{77., 2533.}	125.595	0.886384	newly 5 weeks spec	557.	51.	{0., 62.}	97.1562	2.56201	newly 5 weeks cumul	2471.	1240.78	{77., 3560.}	103.477	1.0255	newly 57 weeks cumul	5430.	1499.51	{77., 4036.}	91.2256	1.21454
37	newly 3 weeks spec	552.	185.13	{2., 301.}	105.466	1.55685	newly 3 weeks cumul	1345.	522.85	{15., 1054.}	90.6892	0.925663	newly 5 weeks spec	542.	73.53	{0., 149.}	93.0978	2.47159	newly 5 weeks cumul	2492.	731.79	{18., 1765.}	92.0084	1.12466	newly 60 weeks cumul	4762.	834.38	{21., 2280.}	89.6212	1.35488
38	newly 3 weeks spec	663.	241.64	{4., 441.}	107.023	1.53978	newly 3 weeks cumul	1606.	628.43	{25., 1488.}	92.0592	0.957759	newly 5 weeks spec	672.	88.81	{0., 284.}	90.0729	2.41205	newly 5 weeks cumul	3051.	886.88	{25., 2253.}	92.213	1.16276	newly 60 weeks cumul	6153.	983.62	{25., 2533.}	88.6306	1.40572
39	newly 3 weeks spec	517.	166.33	{7., 245.}	91.3714	1.05205	newly 3 weeks cumul	1361.	993.09	{152., 1394.}	99.9096	0.643474	newly 5 weeks spec	517.	62.34	{0., 83.}	91.8221	1.6381	newly 5 weeks cumul	2422.	1151.03	{173., 1599.}	93.0673	0.790385	newly 56 weeks cumul	5258.	1340.71	{173., 1816.}	88.5213	1.04039
40	newly 3 weeks spec	579.	176.53	{0., 173.}	95.5078	1.39152	newly 3 weeks cumul	1495.	990.14	{241., 1319.}	93.4007	0.595981	newly 5 weeks spec	560.	53.38	{0., 56.}	93.8179	1.99786	newly 5 weeks cumul	2700.	1141.16	{248., 1432.}	89.8837	0.772313	newly 56 weeks cumul	5592.	1205.92	{251., 1848.}	86.8244	1.01998
41	newly 3 weeks spec	748.	551.24	{100., 963.}	52.4786	0.370992	newly 3 weeks cumul	1534.	3927.76	{1163., 9593.}	171.249	0.370678	newly 5 weeks spec	1008.	156.36	{8., 371.}	84.4881	1.14659	newly 5 weeks cumul	3552.	4359.48	{1294., 10 743.}	67.853	0.283473	newly 64 weeks cumul	5882.	4627.75	{1294., 11 119.}	55.3885	0.343846
42	newly 3 weeks spec	764.	264.35	{4., 604.}	92.0484	1.17829	newly 3 weeks cumul	1835.	1187.43	{24., 2949.}	99.5177	0.904264	newly 5 weeks spec	835.	113.44	{2., 202.}	90.9844	1.59572	newly 5 weeks cumul	3509.	1471.41	{32., 3613.}	92.2967	1.05317	newly 55 weeks cumul	5877.	1698.72	{38., 4069.}	87.9809	1.16995
43	newly 3 weeks spec	917.	216.48	{0., 725.}	80.1112	1.77245	newly 3 weeks cumul	2107.	3542.89	{728., 7634.}	103.578	0.350581	newly 5 weeks spec	846.	67.34	{0., 236.}	93.279	2.49084	newly 5 weeks cumul	3927.	3724.78	{748., 7634.}	60.0448	0.350575	newly 63 weeks cumul	5868.	3863.11	{755., 7663.}	57.8737	0.434108
44	newly 3 weeks spec	531.	93.64	{0., 98.}	92.3315	1.73868	newly 3 weeks cumul	1403.	1175.47	{81., 2474.}	121.111	0.801523	newly 5 weeks spec	507.	53.35	{0., 58.}	96.9645	2.32382	newly 5 weeks cumul	2442.	1290.36	{81., 3732.}	102.247	0.923375	newly 57 weeks cumul	4771.	1542.77	{81., 4307.}	92.03	1.07214
45	newly 3 weeks spec	842.	241.08	{4., 420.}	99.7316	1.54824	newly 3 weeks cumul	1994.	838.34	{65., 2194.}	94.8275	0.973429	newly 5 weeks spec	777.	79.92	{0., 204.}	90.2265	2.34973	newly 5 weeks cumul	3630.	1072.06	{73., 2312.}	91.9328	1.15367	newly 60 weeks cumul	5942.	1175.43	{83., 3713.}	88.0971	1.3118
46	newly 3 weeks spec	429.	150.96	{1., 671.}	96.4522	1.40702	newly 3 weeks cumul	1207.	1237.33	{38., 7656.}	132.743	0.73621	newly 5 weeks spec	442.	26.31	{0., 38.}	96.3733	2.43485	newly 5 weeks cumul	2135.	1321.2	{40., 7893.}	108.283	0.888806	newly 59 weeks cumul	4374.	1387.04	{43., 7904.}	91.2378	1.1019
47	newly 3 weeks spec	432.	180.73	{1., 230.}	110.521	1.51784	newly 3 weeks cumul	1154.	516.93	{15., 769.}	92.273	0.885209	newly 5 weeks spec	487.	74.31	{0., 113.}	94.1047	2.37306	newly 5 weeks cumul	2160.	720.85	{16., 1794.}	92.7838	1.08286	newly 60 weeks cumul	4498.	831.74	{16., 2299.}	89.7563	1.34464
48	newly 3 weeks spec	691.	144.45	{3., 268.}	89.246	1.35476	newly 3 weeks cumul	1624.	1026.24	{90., 1142.}	116.196	0.897293	newly 5 weeks spec	702.	57.71	{0., 63.}	93.8675	1.98763	newly 5 weeks cumul	3044.	1170.42	{92., 1447.}	100.859	1.05337	newly 57 weeks cumul	6315.	1411.34	{92., 1758.}	91.2735	1.23223
49	newly 3 weeks spec	507.	195.44	{1., 696.}	97.5306	1.44657	newly 3 weeks cumul	1374.	1412.67	{29., 7522.}	139.452	0.88774	newly 5 weeks spec	548.	35.53	{0., 40.}	95.8266	2.45102	newly 5 weeks cumul	2534.	1523.18	{35., 7765.}	109.423	1.03761	newly 59 weeks cumul	4591.	1593.7	{40., 7774.}	91.7848	1.19326
50	newly 3 weeks spec	1068.	546.18	{65., 975.}	67.2809	0.598312	newly 3 weeks cumul	2428.	4524.07	{1181., 11 177.}	117.065	0.325358	newly 5 weeks spec	913.	127.99	{2., 355.}	87.0088	1.43421	newly 5 weeks cumul	4440.	4901.64	{1252., 11 376.}	69.2919	0.31771	newly 64 weeks cumul	6234.	5133.98	{1350., 11 394.}	59.6997	0.353968
